# Comparison between intrathecal and intravenous betamethasone for post-operative pain following cesarean section: a randomized clinical trial

**DOI:** 10.12669/pjms.292.2863

**Published:** 2013-04

**Authors:** Taraneh Naghibi, Faramarz Dobakhti, Saideh Mazloomzadeh, Atosa Dabiri, Behnaz Molai

**Affiliations:** 1 Taraneh Naghibi, Department of Anesthesiology and Critical Care Medicine, School of Medicine, Zanjan University of Medical Science, Zanjan, Iran.; 2 Faramarz Dobakhti, School of Pharmacy, Zanjan University of Medical Science, Zanjan, Iran.; 3 Saideh Mazloomzadeh, School of Medicine, Zanjan University of Medical Science, Zanjan, Iran.; 4 Atosa Dabiri, Department of Gynecology and Obstetrics, Zanjan University of Medical Science, Zanjan, Iran.; 5 Behnaz Molai, Department of Gynecology and Obstetrics, School of Medicine, Zanjan University of Medical Science, Zanjan, Iran.

**Keywords:** Intrathecal, Betamethasone, Postoperative pain, Cesarean section

## Abstract

***Objective:*** Inadequate postoperative pain relief after cesarean section can increase complications. In this study, we evaluated the effect of intrathecal betamethasone as an adjunct to bupivacaine on postoperative pain in patients undergoing cesarean section.

***Methodology:*** Ninety-nine patients undergoing cesarean section were assigned to one of three groups. Group 1 (Control) patients received intrathecal bupivacaine, Group 2 patients received intrathecal bupivacaine plus preservative free betamethasone and Group 3 patients received betamethasone intravenously with intrathecal bupivacaine. After surgery, diclofenac in suppository form was administered as needed for analgesia. Postoperative diclofenac requirements, time to first analgesic administration and visual analogue scale pain scores were recorded by a blinded observer.

***Results:*** Supplemental analgesic dose requirement with diclofenac for the first 24 hours were significantly less in both groups that received betamethasone compared to the control group (P <0.0001). The mean duration of postoperative analgesia was 336.8±86 min in Intrathecal group and 312.4±106 min in Intravenous group compared with 245.4±93 min in control group (P =0.001). Visual analogue scale scores were significantly less at 4 hours (P<0.0001) and 6 hours (P<0.0001) after surgery in groups that received betamethasone in comparison to control group. The pain scores at 6 hours after surgery were higher in the Intravenous group compared with the Intrathecal group (P = 0.001); However visual analogue scale was not different at 12 and 24 hours after surgery between groups (p > 0.05).

***Conclusion:*** Intrathecal betamethasone reduced pain and decreased the required dose of diclofenac in 24 hours after cesarean section.

## INTRODUCTION

Inadequate postoperative pain relief after cesarean section can increase health-care costs, delay mother child bonding, increase duration of the hospital stay and reduce patient satisfaction. It can also delay recovery because persistent pain perception can have a negative effect on homeostasis and healing, this is why early treatment of pain with analgesics is important.^1^^,^^2^

Several analgesic techniques have been used to provide postoperative pain relief. Multimodal approaches to postoperative analgesia, with use of different drugs acting via different routes have led to minimal side-effects and good analgesia in the treatment of postoperative pain.^3^

Several investigators have shown steroids analgesic effects. This effect of steroids is caused by inhibition of arachidonic acid that release from damaged cells. Another way to establish analgesia by Steroids is the effect on GABA receptors in supraspinal and spinal sites.^4^ Different methods of steroids administration are parentral, local infiltration at operated site, as an adjuvant in nerve blocks, and central-neuraxial blockade.^3^^,^^5^^-^^7^

Evidence indicates that intrathecal steroids are useful in the treatment of chronic pain^8^^-^^11^ while there are few studies to indicate the effectiveness of intrathecal steroids for the management of postoperative pain up to now. The aim of this study was to assess the analgesic benefits of the intrathecal betamethasone on post operative pain relief after cesarean section. In order to obtain a higher local concentration of drug with few systemic side effects, spinal route was chosen.^4^

## METHODOLOGY

After obtaining approval from the hospital ethics committee and informed consent, ninety-nine patients undergoing cesarean section at our institution were enrolled in this prospective, randomized, double-blinded study. Included were patients with ASA class I and II, age between 20-40 years and scheduled to undergo cesarean section. Patients were excluded if they were receiving analgesics or corticosteroid drugs, there was a contraindication to regional anesthesia, known sensitivity to the study drugs or if they had any history of cardiac, respiratory, neuromuscular, hepatic or renal disease.

Visual analogue scale for pain (VAS-consisting of 100 mm line with 0 = no pain and 100 = the worst possible pain) was explained to all patients during the preoperative visit. In the operating room routine monitors were established (electrocardiogram, non-invasive blood pressure monitor and pulse oximeter). A 16 gauge intravenous canula was sited. An intravenous preload of 5 mL/kg of lactated Ringer’s solution was given before dural puncture. Spinal puncture was performed at the L_3-4 _interspace with 25-gauge Quincke needle through midline approach. The spinal drugs were administered over 30 second.

Patients were randomly allocated to one of three groups: Group 1 (Control) patients received normal saline 0.5 mL intravenously with intrathecal injection of heavy bupivacaine 0.5% 3 mL plus 0.5 mL of 0.9% saline, Group 2 (IT) patients received normal saline 0.5 mL intravenously with intrathecal injection of heavy bupivacaine 0.5% 3 mL plus 0.5 mL preservative free betamethasone (4 mg/mL) and Group 3 (IV) patients received betamethasone 0.5 mL intravenously (4 mg/mL) with intrathecal injection of heavy bupivacaine 0.5% 3 mL plus 0.5 mL of 0.9% saline.

Mean arterial pressure (MAP) and heart rate (HR) were monitored pre and post spinal anesthesia in addition to 10, 20 and 30 min thereafter. Following surgery 100 mg suppository of diclofenac was administered if VAS score was more than 4. Time of the first analgesic (time between intrathecal injection and the first administration of rescue analgesic), as well as the total number of analgesic doses required in the first 24 hours postoperatively, were recorded.

The VAS scores were recorded at 4, 6, 12 and 24 hours from the intrathecal injection. The incidence of postoperative adverse effects (such as nausea, vomiting and headache) in the first 24 hours were evaluated with a “yes” or “no” survey. Nausea was defined as a subjective unpleasant sensation associated with the awareness of the urge to vomit. Vomiting was defined as the forceful expulsion of liquid gastric contents.

Statistical analysis of the data was performed using SPSS 16. The Kolmogorov-Smirnov test was used to evaluate the distribution of variables. Values were expressed as mean ± standard deviation, and number (percentage), as appropriate. Comparisons were performed by one-way analysis of variance (ANOVA) followed by Tukey's Post-hoc test for normally distributed, Kruskal–Wallis test for non-normally distributed, and chi-square test for categorical variables. A *P* value of <0.05 regarded as significant.

## RESULTS

None of the patients was excluded from the study and data of all 99 patients were evaluated. Patient characteristics were comparable (*p*>0.05) with respect to age*, *surgical cause of operation (elective, urgency) and the patients living area (urban, rural) (Table-I).

**Table-I:** Demographic and surgery data

		*Control* *group*	*Intrathecal* *group*	*Intravenous* *group*
Age* (year) *mean ± SD*		26.5±5	25.2±5	25±4
*Surgical cause* ^ ז^ * (n %)*	Elective	11(33.3%)	3(39.4%)	15(45.5%)
	Urgency	22(66.7%)	20(60.6%)	18(54.5%)
Area^ ז^ (n %)	Urban	12(36.4%)	14(42.4%)	14(42.4%)
	*Rural*	21(63.6%)	19(57.6%)	19(57.6%)

*compared with One Way ANOVA test

^ז^ compared with Chi-square test

No statistically significant differences were observed in heart rate and mean arterial blood pressure between the three groups, both preoperatively as well as intraoperatively. There were significant differences in VAS pain score between intrathecal and intravenous groups with control group at 4 and 6 hours post operation. VAS pain score in these hours was significantly lower in these 2 groups than in control group (Table-II). Due to Post Hoc Tukey test, IT group in comparison with IV group had significantly lower VAS at 6 hours after surgery. No statistically significant differences were observed in VAS pain score at 12 and 24 hours post operation between the three groups (Table-II).

**Table-II:** Visual analogue score at various time points postoperatively

	*Control* *group*	*Intrathecal* *group*	*Intravenous* *Group*	p value*
*VAS 4*	6± 2	3.18 ± 1.2	4± 1.3	p<0.0001
*VAS 6*	7.7± 1.8	2.5± 1	4± 1.8	p<0.0001
*VAS 12*	2.4± 2.2	2± 1.6	2.4± 1.7	0.5
*VAS 24*	2.2± 2.2	1.5± 1.5	1.6± 1.5	0.2

*compared with One Way ANOVA test

Values are mean ± SD

On the basis of Kruskal wallis test, Total diclofenac consumption in IT and IV groups were significantly lower than control group at 24 hours post operation. This data was significantly lower in IT group compare to IV group (p=0.014) (Table-III).

Time to first administration of rescue analgesia with diclofenac sodium had statistically significant difference between IT and IV groups with control group (p<0.05). The duration of analgesia in control group was significantly shorter than that in IT and IV groups (Table-III).

**Table-III:** Time to rescue analgesia and analgesic requirement in 24 hours postoperatively

	*Control* *group*	*Intrathecal* *group*	*Intravenous* *Group*	*p value*
*Doses of diclofenac* *requirement in 24 h(mg)*	293.9 ± 82	163.3 ± 82	227.2 ± 103	p<0.0001^×^
*Time to rescue* *analgesic (min)*	245.4 ± 93	336.8± 86	312.4± 106	p=0.001*

*compared with One Way ANOVA test

^×^ compared with Kruskal wallis test Values are mean ± SD

The frequencies of side effects intraoperatively and during the 24 hours observation period were comparable in all three groups. Twenty of 99 patients showed nausea and vomiting intraoperatively. The percentage of patients reporting nausea and vomiting intraoperatively was 25% in Group IT and 15% in Group IV and 60% patients in Control Group, and this result was statistically significant.

In this study, 24 patients reported nausea and vomiting in 24 hours after surgery. The percentage of this complication was 23% in Group IT and 19% in Group IV and 57.1% patients in Control Group, and this result was statistically significant.

The percentage of patients showing headache 24 hours post operation was 33.3% in Group IT and 26.7% in Group IV and 40% patients in Control Group. There were no significant differences in this complication among the study groups. Four patients in Group IT showed agitation and flushing in palmar hands and plantar feet. 

## DISCUSSION

This study demonstrated that 2 mg intrathecal betamethasone is efficacious, practical, and safe for reducing postoperative pain and systemic analgesic drugs requirements, following cesarean section.

The mechanism of analgesic effect of corticosteroids is a subject of debate. Glucocorticoids can block both the cyclo-oxygenase and the lipo-oxygenase pathways in the inflammatory cascade, thus implying a profound effect in the eicosanoid and the prostanoid pain medications.^12^ Johansson et al^13^ found that local methylprednisolone could suppress the nerve transmission in thin unmyelinated C-fibers at 30 min after its application.

Analgesic effects of corticosteroids have been reported after various types of surgery.^12^ Preoperative administration of corticosteroids by oral^8^, intramuscular^14^, epidural^15^ and interscalene block^5^ routes has been shown to reduce overall pain scores and analgesic requirements in the postoperative period without any apparent adverse effects. Although the benefit of administration of betamethasone is commonly accepted, its intrathecal use as an adjuvant to local anesthetic for post operative pain has rarely been described.

Bani-hashem et al^16^ have demonstrated that in 50 patients who were scheduled for orthopedic surgery the addition of intrathecal dexamethasone (8 mg) to bupivacaine (15 mg) significantly improved the duration of sensory block in spinal anesthesia without any changes in onset time and complications.

Taguchi et al^8^ have shown that in 10 patients with cancer pain in whom small-dose of betamethasone was used intrathecally once a week, sufficient pain relief was achieved in about a half of patients and no clinical complications were reported.

Siji et al^15^ reported that 5 mg thoracic epidural dexamethasone reduces pain scores following laparoscopic cholecystectomy. They also found that the cumulative dose of morphine administered over 24 hours was lower in patients that received epidural dexamethasone compared with the control group. Aasboe et al^14^ suggested a single 12 mg dose of IM betamethasone 30 minutes before the start of surgery, could produce analgesic and antiemetic effects after ambulatory surgery. Similarly in our study, VAS pain score at 4 and 6 hours post surgery and diclofenac consumption in IT and IV groups was significantly lower during the first 24 hours after surgery compared to the control group (p<0.05). This indicates the positive effect of betamethasone in reducing postoperative pain. The dose of diclofenac was significantly lower in IT group compared to IV group. Perhaps the reason of this is the direct effect of drugs on nerve fibers. Direct inhibition of prostaglandins and inflammatory mediators with intrathecal steroids in damaged nerves as well as in damaged spinal cord caused more analgesia effect than epidural and systemic steroids. It is compatible with findings of this study in which pain score at 6 hours after surgery in IT group was lower in comparison with other groups.

Kroin et al^17^ have demonstrated that after bilateral foot incision in rats, lumbar spinal COX-2 protein levels increased at 3 and 6 hours after incision while at later times, this protein level shows no difference compared with control group. This is consistent with the findings of the present study in which betamethasone reduced the VAS pain score at 4 and 6 hours after surgery in IT and IV groups. The highest effect of betamethasone correlates with the highest level of COX-2 when COX-2 was inhibited with betamethasone. Whereas at 12 and 24 hours after surgery, when the level of COX-2 was low, the effect of betamethasone was not significant in IT and IV groups in comparison with the control group.

In the present study, betamethasone was chosen based on previous studies which had proven the safety of betamethasone in the intrathecal administration. High water solubility of betamethasone is appropriate for intrathecal administration. This drug has a weak mineralocorticoid activity, with very low sodium and water retention, and high anti-inflammatory effect, which is important for postoperative analgesia.^18^

Langmayr et al^19^ have shown that intrathecal injection of betamethasone in patients following lumbar disc surgery had no significant side effects. Latham et al^20^ in an animal study found that intrathecal administration of 5.7 mg betamethasone in sheep' spinal cord, did not lead to any pathological change. However, higher doses of betamethasone (11.4 mg) caused neurotoxicity, which was dose dependent. Administration of low dose betamethasone (2 mg) in the recent study resulted in no dangerous side effect, too (only four patients in the IT group experienced agitation and transient flushing of palms and feet, for which the reason requires further studies).

Antiemetic mechanism of corticosteroids is not clear. It is probably due to inhibition of prostaglandins, which start the process of vomiting. Previous studies have shown the mechanism of antiemetic effective of corticosteroids is reduction of the serotonin level in the central nervous system and changes in the blood-brain barrier permeability. The center which controls nausea and vomiting is affected by different areas such as CTZ. This area is full of dopamine, muscarine, opioid and serotonin receptors. These receptors have an important role in the transfer of impulses to the vomiting center. Receptors in the CTZ are not the only cause of nausea and vomiting, and roles of other receptors in vomiting have been described.^21^ In this study, nausea and vomiting in the IV group was lower than that of IT and control groups. One possible explanation for this different response can be explained by peripheral antiemetic effect of corticosteroids.

## CONCLUSION

This study shows that intrathecal betamethasone can reduce pain and decrease the required dose of diclofenac in 24 hours after cesarean section.
